# Naringin prevents heart mitochondria dysfunction during diabetic cardiomyopathy in rats

**DOI:** 10.5599/admet.2571

**Published:** 2025-01-25

**Authors:** Ilya B. Zavodnik, Tatsiana A. Kavalenia, Siarhei N. Kirko, Elena B. Belonovskaya, Irina A. Kuzmitskaya, Yulia V. Eroshenko, Elena A. Lapshina, Vyacheslav U. Buko

**Affiliations:** 1 Department of Biochemistry, Yanka Kupala State University of Grodno, Bulvar Leninskogo Komsomola, 5, 230009 Grodno, Grodno, Belarus; 2 Division of Biochemical Pharmacology, Institute of Biochemistry of Biologically Active Compounds, National Academy of Sciences, Bulvar Leninskogo Komsomola, 50, 230030 Grodno, Belarus

**Keywords:** Cardiac mitochondria, flavonoids, respiration, islets

## Abstract

**Background and purpose:**

Cardiac mitochondria dysfunction plays a central pathophysiological role in the abnormal glucose metabolism in the heart during diabetic cardiomyopathy. The present study evaluated the effects of flavonoid glycoside naringin treatment on the interconnection between changes in cardiac mitochondria oxygen consumption, membrane potential and mitochondrial Ca^2+^ sensitivity during type 1 diabetes.

**Experimental approach:**

Type 1 diabetes was induced by an intraperitoneal injection of streptozotocin (40 mg/kg) in rats and islet morphology, glucose and insulin levels, changes in heart mitochondria respiration, membrane potential, spontaneous and Ca^2+^ - induced mitochondrial permeability transition (MPT) pore opening were evaluated.

**Key results:**

Diabetes resulted in typical signs of hyperglycaemia, which were accompanied by a rat cardiac mitochondria dysfunction, manifested by decreased *V*_2_ and *V*_3_ rates of oxygen consumption, while the initial membrane potential value remained unchanged. The rates of Ca^2+^-induced MPT pore opening and Ca^2+^-induced membrane potential dissipation in isolated mitochondria decreased under type 1 diabetes. The naringin treatment (40 mg/kg of the body weight, 4 weeks) partially recovered impaired cardiac mitochondria respiration, decreased spontaneous and increased Ca^2+^-induced MPT pore opening, improved pancreatic islets morphology and dystrophic changes, lowered glycated haemoglobin and blood plasma urea, and decreased the oxidative stress level with glucose or insulin concentrations remaining unchanged in diabetic animals.

**Conclusions:**

Naringin administration demonstrated beneficial effects during type 1 diabetes and represents a promising therapeutic (or nutraceutical) approach for diabetes treatment.

## Introduction

Diabetes, one of the most common metabolic diseases in humans, is a major public health problem, which is provoked by defects in insulin secretion due to pancreatic β-cell destruction (type 1) or by insulin resistance of peripheral tissues (type 2) (or by both) [1]. Animal models of type 1 and type 2 diabetes are widely used to study the mechanism(s) of the onset and progression of diabetes and its complications and to search for new pharmaceuticals for improving the metabolism of lipids and carbohydrates and diabetes treatment. Both type 1 and type 2 diabetes mellitus highly increase the risk of developing cardiovascular diseases such as coronary artery disease and heart failure (the term ‘diabetic cardiomyopathy’) [2]. Diabetic cardiomyopathy is a primary disease in diabetic patients characterized by diastolic dysfunction and abnormal cardiac structure in the absence of other cardiac risk factors. Pathophysiological mechanisms of cardiomyopathy during diabetes are connected with heart metabolic abnormalities, energy production damages, impaired calcium homeostasis and cardiac insulin signalling pathway, increased inflammation and oxidative stress, as well as reduced nitric oxide bioavailability and an accumulation of advanced glycation end products [3], activation of renin-angiotensin–aldosterone system, increased free fatty acid level, development of myocardial fibrosis, abnormalities in AMP-activated protein kinase, peroxisome proliferator-activated receptors, protein kinase C, microRNA, and exosome pathways [4]. Recently, it was established that lipotoxic-related events and increased metabolism of free fatty acids might play a central role in the initiation and progression of human diabetic cardiomyopathy [5].

Mitochondria normally occupy about 20 to 30 % of the total cell volume of cardiomyocytes [6]. Mitochondrial dysfunction plays a central pathophysiological role in abnormal glucose metabolism (a reduction in the myocardium’s ability to use glucose), β-cell dysfunction and insulin resistance [7]. The reviews of Bugger and Abel [2,8] concluded that the heart mitochondrial dysfunction in humans with types 1 and 2 diabetes ultimately led to contractile dysfunction. The mechanisms of cardiac mitochondrial impairments during type 2 diabetes are: lipid product accumulation and fatty acid-induced mitochondria uncoupling, mitochondrial reactive oxygen species (ROS) generation, mitochondrial proteomic remodelling, Ca^2+^-handling impairments, mitochondrial biogenesis alteration, decreased mitochondrial content, mitochondrial permeability transition [2], reduced cardiac phosphocreatine/ATP ratio and enhanced ATP utilization [9]. The important questions about whether mitochondrial dysfunction is a cause or a consequence of diabetes remain unanswered [10,11]. Similarly, the rates of the long-chain fatty acid oxidation are markedly increased in heart mitochondria of streptozotocin (STZ)-diabetic (type 1) rats, revealing a striking expression of mitochondrial long-chain fatty acid transport and oxidation genes, accompanying overexpression of acyl-CoA hydrolysing enzyme and uncoupling protein UCP-3 [12]. The genes involved in oxidative phosphorylation (OXPHOS) exhibit reduced expression in the skeletal muscle of diabetic and prediabetic humans [13]. Krako Jakovljević *et al*. [10] suggested that mitochondrial respiratory capacity decreases in the liver, muscle, blood mononuclear cells and platelets during type 2 diabetes progression and that, consequently, mitochondria are the target of pharmacological intervention in diabetes (OXPHOS modulators, AMPK activators, PPAR agonists, antioxidants, mitochondrial permeability transition (MPT) pore inhibitors). On the other hand, islet mitochondria dysfunction and structural changes (swelling of mitochondria, decreased ATP level, increased uncoupling protein 2 expression) play a significant role in pancreatic β-cell failure and diabetes progression [14,15].

The flavonoid naringin, the main polyphenolic component of citrus fruits (naringenin is naringin aglycon), present in human diet, has been found to exert anticarcinogenic, cardioprotective, anti-inflammatory, anti-apoptotic, anti-proliferative, and antimutagenic effects through various molecular mechanisms: by the reduction in oxidative stress, modulation of cell signalling responses, regulation of certain genes expression, direct interaction with cell and cellular components [16,17]. At the same time, the oral bioavailability of naringin in humans has been reported to be only 5 to 9 % [18]. It was established that naringin reduces calpain activity, TNF-α, and IL-6 levels and decreases NF-κB expression, thereby helping to avoid cardiomyopathy in type 2 diabetic mice [19]. However, the potential specific cardioprotective mechanisms of naringin during type 1 diabetes remain unclear.

The present study was aimed at evaluating the effects of naringin treatment on the interconnection between changes in cardiac mitochondria oxygen consumption, membrane potential and mitochondrial Ca^2+^ sensitivity associated with a risk of cardiovascular complications during experimental type 1 diabetes. We hypothesized that mitigation of cardiac mitochondria dysfunction and Ca^2+^ sensitivity by the flavonoid naringin could have a beneficial effect during type 1 diabetes in the correction of mitochondria-related complications. Further insight into the potential of natural compounds, naringin among them, in improving the impaired heart mitochondria functions associated with a risk for cardiovascular complications in diabetes is an important task.

## Experimental

### Reagents

Naringin (4’,5,7-trihydroxy-flavanone 7-rhamnoglucoside), streptozotocin (STZ), trichloroacetic acid, calcium chloride dehydrate, succinic acid disodium salt hexahydrate, sucrose, ethylene glycol-bis(β-aminoethyl ether)-N,N,N′,N′-tetraacetic acid tetrasodium salt (EGTA), carbonyl cyanide *p*-trifluoromethoxyphenyl hydrazone (FCCP), safranin O, bovine serum albumin fraction V (BSA), ethylenediaminetetraacetic acid disodium salt (EDTA), 4-(2-hydroxyethyl)piperazine-1-ethanesulfonic acid (HEPES), cyclosporin A, adenosine 5′-diphosphate sodium salt (ADP), ethanol, and other chemicals were purchased from Merck / Sigma-Aldrich (St Louis, MO, USA, or Steinheim am Albuch, Germany). All solutions were made with water purified in the Milli-Q Direct system (Merck KGaA, Darmstadt, Germany). Organic solvents were of analytical grade and used without further purification.

### Animal experiment

We used male albino Wistar rats with a starting weight of 210 to 230 g. The animals had free access to a standard balanced diet and tap water and were kept under normal conditions (a 12-hour light/darkness cycle at 22±2 °C and 55±5 % relative humidity). The animals were divided into three groups of 10 rats each. Two groups of the animals were treated with a single intraperitoneal injection of STZ (40 mg/kg body) dissolved in 0.1 M Na-citrate buffer, pH 4.5, after 12-hour starvation. Only rats with blood glucose concentrations of over 15.0 mmol/l (Blood Glucose Sensor Electrodes, MediSense, Abbot Laboratories, UK) after the STZ treatment were used in the study. Control animals received a single intraperitoneal injection of the same volume of the Na-citrate buffer. After two weeks of acclimatization, half of the STZ-treated rats were given naringin daily (40 mg/kg body) in physiological saline intragastrically between 10 and 11 a.m. for 4 weeks. This dose was selected based on the previous studies [20]. The control and diabetic groups without the naringin treatment received an equivalent amount of saline daily, intragastrically. Rats were sacrificed under anaesthesia after 4 weeks of naringin (or saline) treatment. Blood samples were drawn by an abdominal aorta puncture. The heart and pancreas were excised immediately from each animal. Heparinized blood samples were centrifuged at 1000 rpm for plasma separation. Blood plasma was placed in an ice bath and used for further measurements.

The care, use, and all procedures performed on rats were approved by the Ethic Committee of the Institute of Biochemistry of Biologically Active Compounds of the National Academy of Sciences of Belarus, Grodno (Protocol No 29/20 of 23.05.2020) and complied with the European Convention for the Protection of Vertebrate Animals Used for Experimental and Other Scientific Purposes and the Guide for the Care and Use of Laboratory Animals [21].

### Isolation of rat heart mitochondria

The coupled myocardium mitochondria were isolated from the rat heart by differential centrifugation [22,23]. We used the isolation medium containing 0.25 M sucrose, 0.005 M HEPES, 0.0002 M EDTA, and 0.1 % BSA, pH 7.4. The isolated heart was dried on filter paper, quickly transferred into an ice-cold 0.9 % KCl solution and carefully washed from the blood. The muscle tissue was weighed, crushed with scissors on ice and homogenized using a glass homogenizer with a Teflon pestle in the isolation medium at 4 °C. The nuclear fraction was removed by centrifugation at 650 g (10 min, 4 °C) (a Hermle Z 32 HK centrifuge, Hermle Labortechnik GmbH, Germany). The mitochondria were sedimented by centrifugation at 8,500 *g* (10 min, 4 °C) and washed two times with the isolation medium. The pellet of mitochondria was resuspended to an approximate protein concentration of 25-30 mg/ml in the isolation medium. Protein concentrations in myocardium mitochondria were determined by the method of Lowry *et al.* [24].

### Histological studies of the pancreas

The excided pancreas was fixed in a 10 % neutral formaldehyde solution, dehydrated in a graded series of ethanol concentrations and embedded in paraffin. 5-μm sections were cut and stained with haematoxylin and eosin. Staining with aldehyde and fuchsin was used for selective detection of pancreatic β-cells. Tissue sections were imaged with an Olympus CX-41 light microscope using a 40x objective, and the digital images were captured with an Olympus C-5060 camera (Olympus, Tokyo, Japan). Analyses were performed using Image J morphometric analysis software (NIH, USA). The point counting method was used for measurements of islet size and islet cell counting [25]. The formula of Fullman was applied to calculate the mean islet diameter, mean area and mean islet volume [26].

### Measurements of cardiac mitochondrial respiration and membrane potential

Respiration parameters of isolated cardiac mitochondria were measured using an oxygen Clark-type electrode (Hansatech Instruments Limited, Great Britain) in the medium containing 0.25 M sucrose, 5 mM KH_2_PO_4_, 5 mM HEPES, 10 M KCl, 2 mM MgCl_2_, 0.2 mM EGTA, рН 7.4, at 26 °C. The mitochondrial suspension (0.5 mg protein/ml) in the respiratory buffer was continuously stirred. To study FADH_2_-dependent respiration, the oxygen consumption rates were determined in the presence of 5 mM succinate as a substrate (respiratory state 2, *V*_2_), after ADP (200 μM) addition (ADP-stimulated state 3, *V*_3_) and after ADP consumption (state 4, *V*_4_). The acceptor control ratio (ACR) equal to the ratio of the respiratory rates (*V*_3_/*V*_2_) of mitochondria in state 3 and state 2, the respiratory control ratio (RCR) equal to the ratio of the respiratory rates (*V*_3_/*V*_4_) of mitochondria in state 3 and state 4, and the coefficient of phosphorylation (ADP/O ratio) equal to the ratio of the consumed ADP to the consumed oxygen atoms were calculated.

The mitochondrial membrane potential (0.3 mg of protein/ml) was detected using a Perkin-Elmer LS55 spectrofluorometer (Great Britain) as the changes in the fluorescent intensity of the dye safranin O (8 μM) at *λ*_ex_/*λ*_em_ = 495/586 nm [27,28] in the medium containing 0.25 М sucrose, 5 mМ HEPES, 5 mM KH_2_PO_4_, 2 mM MgCl_2_, 0.05 mM EGTA, and 5 mM succinate as a substrate, рН 7.4, at 26 °С. The positively charged dye accumulated in mitochondria depending on the mitochondrial membrane potential, resulting in intramitochondrial dye fluorescence quenching. Complete depolarization of mitochondria to calibrate the dye fluorescence was achieved by the addition of the uncoupler FCCP (0.5 μM). For membrane potential characterizing, we presented parameters [*I*_0_ per mg protein] and [(*I*_FCCP_ - *I*_0_) per mg protein], where *I*_0_ is initial safranin O fluorescence intensity in mitochondrial suspension, and *I*_FCCP_ is safranin O fluorescence intensity after FCCP addition. The value [(*I*_FCCP_ - *I*_0_) per mg protein] is proportional to the capacity of the mitochondria to accumulate dye (initial membrane potential). The effect of Ca^2+^ - ions on membrane potential was measured as the fluorescence change after the addition of calcium ions (concentration of free Ca^2+^ = 550 μM) and was presented as [(*I*_FCCP_ – *I*_Ca_) per mg protein). This value is reversible to the mitochondria sensitivity to Ca^2+^.

### Mitochondrial swelling determination

Са^2+^-induced swelling of respiring cardiac mitochondria was measured as we described earlier [29]. Briefly, the extent of the mitochondrial membrane transition (MPT) pore formation was determined from the changes in the optical density of mitochondrial suspension at 540 nm and 26 °С using the buffer containing 120 mM KCl, 5 mМ HEPES, 5 mМ KH_2_PO_4_, 2 mM MgCl_2_, 0.05 mM EGTA, pH 7.4. Isolated mitochondria (0.5 mg of protein/ml) were added to the medium containing respiratory substrate (5 mM succinate). After 5 min of incubation, Ca^2+^ ions (free Ca^2+^ = 550 μM) were added and the rate (Δ*D*^540^ / min^-1^) of the termination phase of swelling was measured using a Jasco V-650 UV-VIS spectrophotometer (Japan). At the end of the measurements, the uncoupler FCCP (0.5 μM) was added to the mitochondria to control the completion of the process of MPT pore formation.

### Blood biochemical parameters

Fasting glucose concentration in the tail vein blood was measured twice per week. The serum level of fasting insulin was measured by using a Rat INS (Insulin) ELISA commercial Kit from Wuhan Fine Biotech Co., Ltd (China). Total and glycosylated haemoglobin (HbA1c), containing 1-deoxy-1(N-valyl)fructose, total plasma protein, albumin, and urea contents in blood serum were measured using commercial kits from AnalizMedProm (Minsk, Belarus). Homeostasis model assessment of insulin resistance (HOMA-IR) was calculated using the formula: [fasting plasma glucose (mmol/l)/× fasting serum insulin (mU/l)] / 22.5; higher HOMA-IR values show lower insulin sensitivity (insulin resistance).

The accumulated products of membrane lipid peroxidation (thiobarbituric acid-reactive substances) in kidney tissue and erythrocytes were monitored according to the method of Stocks and Dormandy, assuming that the molar absorption coefficient was *ε*_532_=0.156 μM^-1^ cm^-1^ [30]. Reduced glutathione GSH (non-protein thiols) in kidney tissue and erythrocytes was assayed with Ellman's reagent using the molar absorption coefficient *ε*_412_=13.6 mM^-1^ cm^-1^ [31]. Glutathione peroxidase activity in erythrocytes was determined by the rate of GSH oxidation according to the method of Martinez *et al.* [32].

### Statistics

The data of the experiments were processed statistically by the package of the applied program Statistica 10.0 and presented as a mean ± standard error. The normality of distribution was assessed by the Shapiro-Wilk’s test. The reliability of differences between the parameters was analysed using the non-parametric Mann-Whitney U test and the Kruskal-Wallis H test when the data distribution was not the normal distribution, and the standard Student t-test for the data showed no deviations from normality. The level of significance was considered at *p* <0.05.

## Results

### Blood plasma parameters of rats during diabetes and naringin treatment

As a result of STZ injection in rats, we observed the typical signs of hyperglycaemia: the blood glucose and glycated haemoglobin levels significantly increased, and insulin content and animal body mass considerably decreased (Table 1).

**Table 1.** Fasting blood glucose, total and glycated haemoglobin, insulin, urea, total plasma protein, and albumin levels, and animal body mass, HOMA-IR value, and relative kidney mass in diabetic rats treated with naringin for four weeks.

Parameter	Control	Diabetes	Diabetes+naringin
Content of blood glucose at the onset of the experiment, mmol / l	5.05 ± 0.58	18.97 ± 1.77^a^	17.32 ± 1.83^a^
Content of blood glucose at the end of the experiment, mmol / l	5.35 ± 0.63	25.03 ± 2.98^a^	19.27 ± 2.58^a^
Content of total haemoglobin, g / l	49.47 ± 3.40	37.22 ± 4.34^a^	42.43 ± 5.26^a^
Content of glycated haemoglobin, %	4.50 ± 0.58	14.87 ± 1.92^a^	8.06 ± 2.17^ab^
Amount of insulin, mU / l	16.06 ± 1.58	9.91 ± 1,09^a^	10.79 ± 1.41^a^
HOMA-IR value	3.6 ± 0.50	11.12 ± 1.38^a^	9.34 ± 1.46^a^
Content of urea, mmol / l	5.62 ± 0.54	23.45 ± 1.96^a^	14.04 ± 1.62^ab^
Content of total plasma protein, g / l	97.7 ± 7.6	123.8 ± 9.4^a^	99.2 ± 8.0^b^
Content of albumin, g / l	43.3 ± 5.5	36.5 ± 4.8	39.8 ± 3.8
Relative kidney mass, g / 100 g of body weight	0.26±0.03	0.43±0.04^a^	0.40±0.02^a^
Body mass at the onset of the experiment, g	235 ± 20	215 ± 15	220 ± 15
Body mass at the end of the experiment, g	240 ± 15	195 ± 20^a^	210 ± 10^a^

The results are shown as the mean ± standard error.

^a^*p* < 0.05 compared to the control group;

^b^*p* < 0.05 compared to the diabetes group.

The HOMA-IR value increased, demonstrating elevated systemic insulin resistance. Treatment of diabetic animals with naringin (4 weeks) did not influence the HOMA-IR value or body mass but decreased the glycated haemoglobin content with statistical significance. The blood glucose content slightly decreased and the insulin level slightly increased under flavonoid administration, but these changes were not statistically significant in our experiment. The level of total haemoglobin was decreased in the STZ-treated animals as a sign of anaemia and did not change after naringin administration (Table 1).

The content of blood urea and total plasma protein was significantly higher in the diabetic group as compared to the control as a result of nephrotic impairments and the 4-week naringin administration improved blood plasma urea and protein contents (Table 1). The naringin administration did not change either the content of blood plasma albumin or the animal body mass (Table 1). The relative kidney mass value was significantly higher in the diabetes group than in the control animals. The level of lipid peroxidation products increased and the reduced glutathione level decreased (we measured the oxidative stress markers in kidney tissue). Naringin administration partially prevented TBARS accumulation, but not GSH oxidation, in the kidney (Table 2).

**Table 2.** Lipid peroxidation products and reduced glutathione levels in rat kidney and erythrocytes, erythrocyte glutathione peroxidase activity in diabetic rats treated with naringin for four weeks

Parameter	Control	Diabetes	Diabetes+naringin
Kidney TBARS content, nmol / g kidney tissue	31.8±5.2	49.9±4.5^a^	42.2±4.4^ab^
Kidney GSH content, nmol / g kidney tissue	12.8±1.1	8.9±1.0^a^	9.7±0.8^a^
Erythrocyte TBARS content, nmol / ml packed cells	3.96±0.54	5.23±0.67^a^	4.08±0.56^b^
Erythrocyte GSH concentration, mM	1.83±0.21	1.41±0.17 ^a^	1.64±0.15
Erythrocyte glutathione peroxidase activity, (μmol / min) / ml packed cells	597±65	433±50^a^	512±10

The results are shown as the mean ± standard error.

^a^*p* <0.05 compared to the control group;

^b^*p* <0.05 compared to the diabetes group.

Table 2 presents an increase in lipid peroxidation level and a decrease in glutathione level and glutathione peroxidase activity in rat erythrocytes during diabetes. Naringin administration partially recovered these parameters to the control values.

### Histology of pancreatic islets during diabetes and naringin treatment

**Figure 1.**
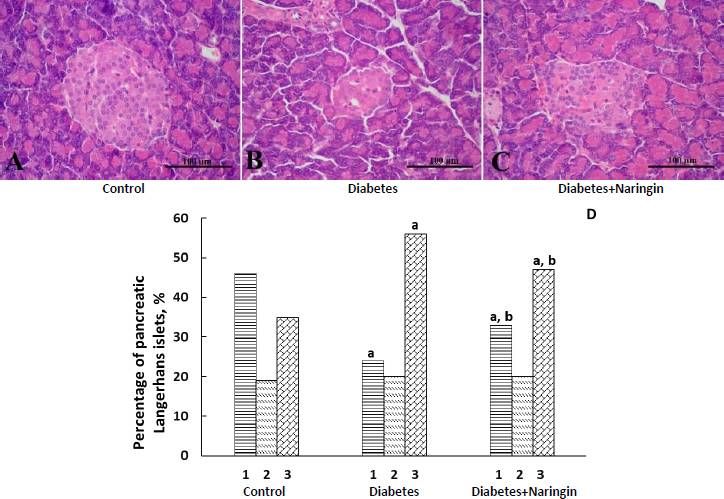
The histological structure of the pancreas from rats with SZT-induced diabetes: effect of naringin treatment (representative haematoxylin and eosin-stained sections). (A) control (normal Langerhans’s islet); (B) diabetes (dystrophic changes in Langerhans islet and vacuolization of secretory cells); (C) diabetes after naringin treatment (improvement of islet histopathology, increase in the number of secretory cells, decrease in the dystrophy degree). Each scale bar indicates 100 μm; (D) Percentage of the pancreatic Langerhans islets of the corresponding area in control, diabetic and naringin-treated diabetic rats: 1 - more than 10001 μm^2^, 2-2501 to 10000 μm^2^, 3-501 to 2500 μm^2^. ^a^*p* <0.05 as compared to the control group; ^b^*p* <0.05 as compared to the diabetes group

The histological evaluation of the haematoxylin and eosin-stained control pancreatic sections showed a typical histological structure of the pancreas without destructive changes and inflammation. The Langerhans islets were predominantly medium and large, clearly defined, surrounded by a thin layer of connective tissue (Fig. 1A). The analysis of the pancreas from diabetic group rats showed considerable islet impairments: shrinkage in the islet sizes, disrupted islet architecture and destruction of β-cells (Fig. 1B). The diabetic animals exhibited β-cell atrophy and degranulation with pronounced vacuolation and pycnotic nuclei (Fig. 1B). Simultaneously we observed significant lymphocyte infiltration. The area, the volume and the diameter of the pancreatic islets were markedly lowered in the STZ-treated animals in comparison with the control group (Table 3).

**Table 3.** Histomorphometric parameters of pancreatic islets from control, SZT-treated diabetic rats, effect of naringin treatment

	Control	Diabetes	Diabetes + naringin
Area of islets, μm^2^	14060 ± 730	5990 ± 490^a^	8180 ± 650 ^ab^
Diameter of islets, μm	138 ± 12	80 ± 7 ^a^	99 ± 9^ab^
Volume of islets, μm^3^	(16.6±1.5)×10^5^	(4.5±0.6)×10^5 a^	(6.2±0.6)×10^5 ab^

^a^*p* <0.05 as compared to the control group;

^b^*p* <0.05 as compared to the diabetes group.

Naringin administration increased β-cell granulation and lowered vacuolation in many islets as compared to the STZ-treated group (Figure 1C), elevated the number of pancreatic β-cells, as well as increased the area, diameter and volume of the islets in comparison with the untreated diabetic group (Table 3).

At the same time, we observed signs of dystrophy and inflammation in the pancreas of the animals of this group (Figure 1C). Figure 1D demonstrates the distribution of islets, depending on the size, in animals of different experimental groups. The number of large-size islets decreased with the increase in the number of small-size islets during diabetes, and treatment with naringin elevated the number of large-size islets.

### Cardiac mitochondria dysfunction during diabetes and naringin treatment

**Figure 2.**
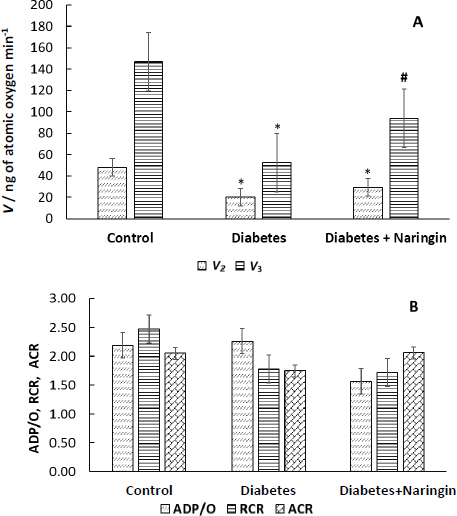
Respiratory parameters of rat cardiac mitochondria during diabetes and naringin treatment: A - the oxygen consumption rates *V*_2_ and *V*_3_ (rates expressed as ng atomic oxygen/min), *V*_2_ is the respiration rate in the presence of the substrate, *V*_3_ is the ADP-stimulated respiration rate; B - the phosphorylation coefficient (ADP/O ratio), RCR equal to the ratio of the respiratory rates (*V*_3_/*V*_4_), ACR equal to the ratio of the respiratory rates (*V*_3_/*V*_2_); 250 mM sucrose, 5 mM KH_2_PO_4_, 5 mM HEPES, 10 mM KCl, 2 mM MgCl_2_, 0.2 mM EGTA, рН 7.4, at 26 °C, 0.5 mg protein/ml, 5 mM succinate as a substrate, 200 μM ADP. *p<0.05 in comparison with the control group, ^#^*p*<0.05 in comparison with the diabetes group

The numerous abnormalities in the heart mitochondria functional activities during diabetes are widely known. In our experiment, the STZ injection to the rats considerably impaired the rates of oxygen consumption *V*_2_ and *V*_3_ by isolated cardiac mitochondria, energized by succinate (Figure 2A), while the parameters ACR, RCR and the ADP/O ratio were not changed appreciably in the animals after 4-week diabetes (Figure 2B). The intake of the flavonoid naringin by diabetic animals partially normalized the rate of O_2_ consumption *V*_3_ by heart mitochondria but did not considerably affect the ADP/O, ACR and RCR coefficients in the diabetic rats (Figure 2).

Using the potential sensitive probe safranin O-loaded mitochondria, we evaluated the effect of diabetes and naringin treatment on the rat cardiac mitochondria membrane potential. Simultaneously, we analysed the mitochondria sensitivity to Ca^2+^ ions.

**Figure 3.**
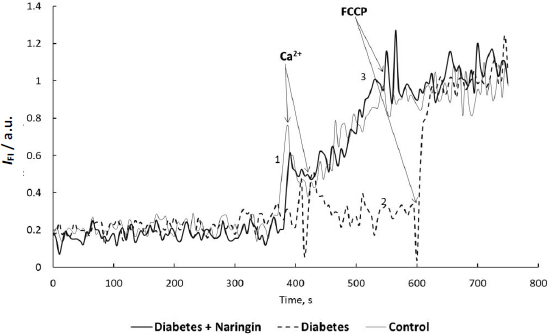
The representative tracks of safranin O (8 μM) fluorescence intensity in cardiac mitochondria suspension (0.3 mg of protein/ml) loaded with the probe under addition of Ca^2+^ ions (concentration of free Ca^2+^ = 550 μM) and the uncoupler FCCP: control group (1), diabetes group (2), diabetes + naringin group (3). The fluorescence intensity of the dye was measured at *λ*_ex_/*λ*_em_ = 495/586 nm in the medium containing 0.25 М sucrose, 5 mМ HEPES, 5 mM KH_2_PO_4_, 2 mM MgCl_2_, 0.05 mM EGTA and 5 mM succinate as a substrate, рН 7.4, at 26 °С.

Figure 3 shows the representative tracks of the fluorescence intensity of the safranin O under the addition of Ca^2+^ ions and the uncoupler FCCP. Diabetes considerably decreased the depolarization effect of Ca^2+^ ions (concentration of free Ca^2+^ = 550 μM, added Ca^2+^ free for a given Ca^2+^ total was determined using the Ca-EGTA online calculator) on the membrane potential, measured as the fluorescence intensity of the probe-loaded mitochondria.

**Figure 4.**
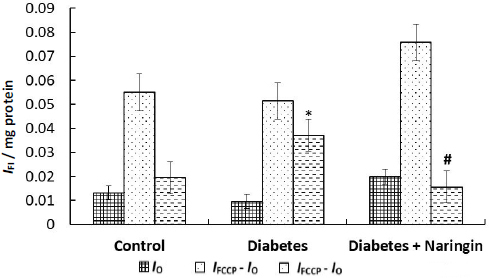
The initial safranin O fluorescence intensity (*I*_0_), fluorescence intensity after uncoupler FCCP addition to the suspension (*I*_FCCP_ - *I*_0_) and changes of the fluorescence intensity after Ca^2+^-ions addition (*I*_FCCP_ - *I*_Ca_] to the suspension of isolated heart mitochondria of animals of control, diabetes and diabetes+ naringin groups. The fluorescence intensity of the dye was measured at *λ*_ex_/*λ*_em_ = 495/586 nm in the medium containing 250 mМ sucrose, 5 mМ HEPES, 5 mM KH_2_PO_4_, 2 mM MgCl_2_, 0.05 mM EGTA and 5 mM succinate as a substrate, рН 7.4, at 26 °С. *p <0.05 as opposed to the control group, ^#^*p* <0.05 as opposed to the diabetes group

As Figures 3 and 4 showed, the initial safranin O fluorescence intensity, as well as the fluorescence intensity after uncoupler FCCP addition to the isolated heart mitochondria suspension, were similar in all rat groups.

**Figure 5.**
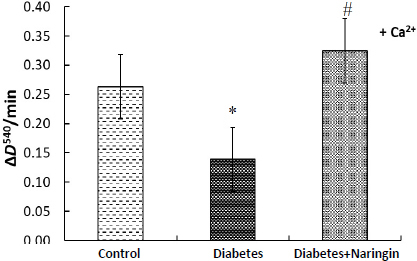
Rates of MPT pore formation (Δ*D*^540^/min) induced by calcium ions (concentration of free Ca^2+^ = 550 μM) in isolated cardiac mitochondria of different animal groups *in vitro*. The changes in the optical density of mitochondrial suspension (0.5 mg of protein/ml) at 540 nm and 26 °С were registered in the medium 120 mM KCl, 5 mМ HEPES, 5 mМ KH_2_PO_4_, 2 mM MgCl_2_, 0.05 mM EGTA, pH 7.4, 5 mM succinate. *p <0.05 in comparison with the control group, ^#^*p*<0.05 in comparison with the diabetes group.

One can conclude that the initial cardiac mitochondria membrane potential values did not change during diabetes and naringin administration. At the same time, the membrane potential changes after the addition of calcium ions to the mitochondria of diabetic animals were considerably higher ((*I*_FCCP_ - *I*_0_) per mg protein was smaller) in comparison with control rats and diabetic animals after naringin treatment (Figures 3 and 4), demonstrating changes in susceptibility of cardiac mitochondria to Ca^2+^ during diabetes and polyphenol treatment. The mitochondria exposure to excess Ca^2+^ ions resulted in MPT pore formation. Figure 5 shows the rates of MPT induced by calcium ions (concentration of free Ca^2+^ = 550 μM) in isolated cardiac mitochondria of different animal groups *in vitro*. (The MPT was prevented by CsA pretreatment.)

**Figure 6.**
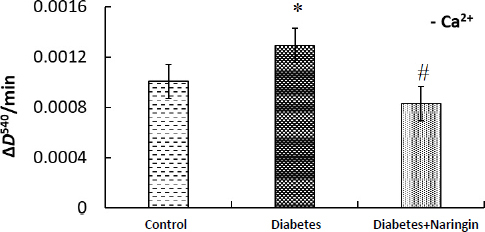
Rates of spontaneous MPT pore opening (Δ*D*^540^ / min) (in the absence calcium ions) in isolated cardiac mitochondria of different animal groups *in vitro*. The changes in the optical density of mitochondrial suspension (0.5 mg of protein per ml) at 540 nm and 26 °С were registered in the 120 mM KCl, 5 mМ HEPES, 5 mМ KH_2_PO_4_, 2 mM MgCl_2_, 0.05 mM EGTA, pH 7.4, 5 mM succinate. *p <0.05 in comparison with the control group, ^#^*p* <0.05 in comparison with the Diabetes group.

The susceptibility to calcium-induced swelling of the cardiac mitochondria significantly changed during diabetes and naringin administration. Four-week diabetes decreased the sensitivity of cardiac mitochondria to Ca^2+^ ions-induced MPT (decreased the rate of MPT pore formation) and the treatment of diabetic rats with naringin (40 mg/kg) increased the rate of Ca^2+^-induced swelling *in vitro*. Figure 6 demonstrates an increased rate of isolated diabetic cardiac mitochondria spontaneous swelling (without the addition of exogenous calcium) in comparison with control mitochondria. Rates of spontaneous MPT pore opening in heart mitochondria of naringin-treated diabetic animals and control animals were similar.

## Discussion

Heart failure is a multifactorial disease in both type 1 and type 2 diabetes mellitus, resulting in ischemic events and altered vascular permeability [33]. There are numerous hypotheses dealing with the association of types 1 and 2 diabetes with mitochondrial dysfunction and impaired energy metabolism. Mitochondrial dysfunction and complex metabolic changes in the type 2 diabetic human myocardium have been observed earlier in many investigations: increased oxidative stress and mitochondrial network fragmentation in right atrial tissue, more susceptibility to Ca^2+^-induced MPT pore opening and further activation of the intrinsic apoptosis pathway, decreased expression of mtDNA encoded genes, decreased ETC complex activities, increased rate of palmitate oxidation (for review see [34]). Using the permeabilized cardiac myofibers, Anderson et al. directly demonstrated impaired mitochondrial respiratory capacity in the atrial tissue of type 2 diabetic patients, increased mitochondrial H_2_O_2_ production, and reduced glutathione depletion [35]. Another work of these authors showed the lack of differences in respiratory capacity or mitochondrial enzyme content in the hearts of diabetic and nondiabetic patients [36]. Simultaneously, it was suggested that increased myocardial cell death in diabetic patients may be mitochondrial-mediated and that mitochondrial-specific oxidative stress/redox imbalance may play a crucial role in these processes [36].

In addition, calcium handling machinery is directly compromised, as has been found in several animal models of both type 1 and type 2 diabetes. Hyperglycaemia correlates with decreased sarcoplasmic reticulum Ca^2+^ loading, enhanced spontaneous calcium release from the sarcoplasmic reticulum, and reduced cytoplasmic Ca^2+^ clearance [37]. Phenotypic differences can be observed between diabetic cardiomyopathy in type 1 and type 2 diabetes mellitus and between the basis for mitochondrial dysfunction in insulin-responsive type 1 versus insulin-resistant type 2 diabetic hearts, possibly due to different myocardial insulin action [38-40]. It was shown that the ADP-stimulated mitochondrial oxygen consumption and ATP synthesis were decreased, whereas mitochondria demonstrated abnormal morphology without evidence of oxidative stress and any changes in ADP/O ratio and proton leak in type 1 diabetic Akita mouse hearts [39]. Mitochondrial ROS production was increased in the hearts of type 2 diabetic animals, while type 1 diabetic models showed no enhanced production of ROS originating from mitochondria [41]. In the opposite, recently, it was shown that STZ-induced type 1 diabetes displayed oxidative stress, perturbations in Ca^2+^ handling, apoptotic cardiomyocyte death, hypertrophy and fibrosis [40]. Herlein *et al.* [41] showed that STZ-induced diabetic heart and muscle mitochondria were more coupled than control despite a 2- to 4-fold increase in uncoupling protein-3 content, did not generate excess superoxide and showed adaptation to *in vivo* oxidative stress. Fatty acid-induced mitochondrial uncoupling is another trait of type 2 diabetic hearts that does not seem present in type 1 diabetic model hearts [39].

Our experimental type 1 diabetes model showed an animal body mass decrease, kidney nephropathy, lipid peroxidation products accumulation and GSH depletion in kidney tissue and erythrocytes, glutathione peroxidase inhibition in erythrocytes confirmed the role of oxidative stress in diabetic complications. Our morphometric studies revealed a dramatic decrease in the amount of Langerhans islets in the diabetic group as compared to the control animals. β-cells of the diabetic animals exhibited atrophy, degranulation and necrotic changes. We observed marked hyperglycaemia and glycated haemoglobin accumulation during 4-week diabetes, decreased insulin level in the blood plasma, increased insulin resistance (the HOMA-IR value), anaemia and hyperproteinaemia. The administration of naringin to the diabetic animals demonstrated some beneficial effects, partially prevented diabetic complications in cardiac mitochondria and did not influence animal and kidney mass. Naringin treatment reduced the severity of degenerative changes and inflammation in the pancreas of the diabetic rats and increased the amount and the size of the islets and normalized some blood biochemical parameters: decreased the level of glycated haemoglobin and the content of urea and total protein in the blood (prevention of the kidney functional alteration). Earlier, it was shown *in vitro* that a number of polyphenols as reducing agents (flavonoids being among them) inhibited the glycation reaction and enrichment of the diet with natural anti-glycating agents may attenuate protein glycation [42]. In our experiment, the lowering of glucose level and increase in insulin level in the diabetes + naringin group were not statistically significant. Earlier, it was shown that in a manner resembling insulin naringin regulates lipoprotein production and insulin sensitivity in mice with diet-induced insulin resistance *in vivo* [43]. Similarly, a number of previous works showed the putative antihyperglycemic and antioxidant effects of naringenin and its 7-O-beta-D-glucoside in streptozotocin-induced diabetic rats. Intraperitoneal treatment of rats with the flavonoids significantly reduced the levels of blood glucose, glycosylated haemoglobin, and total lipids, increased the level of serum insulin, and protected pancreatic tissue [44,45].

The naringin administration suppressed lipid peroxidation processes (decreased the level of TBARS, but not the concentration of GSH) in kidney tissue. Similarly, the alterations in TBARS and glutathione levels and glutathione peroxidase activity in rat erythrocytes under diabetes and flavonoid administration confirmed the antioxidative properties of naringin. Earlier, we observed an increase in the content of the end-products of lipid peroxidation and oxidatively modified proteins and a decrease of the antioxidative potential of red blood cells in experimental type 1 STZ-diabetic rats and showed that red cabbage anthocyanins reduced the oxidative stress [46].

In our experiment, the respiratory activity of heart mitochondria decreased during type 1 diabetes without changing the efficiency of oxygen consumption (ADP/O coefficient), and naringin partially improved the rate of oxygen consumption. We showed that the heart mitochondria membrane potential did not change markedly during diabetes and naringin treatment. However, the susceptibility of cardiac mitochondria to the depolarizing effect of Ca^2+^ decreased under diabetes. Naringin administration recovered it. We compared the rates of spontaneous and Ca^2+^-induced heart mitochondria swelling for control, diabetic and naringin-treated diabetic rats (Figs. 5 and 6). We demonstrated an increased rate of isolated diabetic cardiac mitochondria swelling (without adding exogenous calcium) and a decreased rate of Ca^2+^-induced MPT pore opening compared with control mitochondria. Similarly, calcium-induced dissipation of membrane potential was lower in diabetic mitochondria than in control organelles. The naringin administration brought these parameters back to the control value. We could suggest that calcium uptake in mitochondria under diabetes was disturbed and naringin recovered the altered mitochondrial sensitivity to calcium (due to ionophoric properties) during 1 type diabetes. Earlier, using electron microscopy, Shen *et al.* [47] showed severe damage to cardiac mitochondria in an OVE26 mouse model of type 1 diabetes: the mitochondrial area and the number significantly increased, indicating impaired mitochondrial function and stimulated mitochondrial biogenesis. These transformations were accompanied by alteration of a number of heart mitochondrial proteins, reduced state 3 respiratory rate, and increased oxidative stress [47].

In comparison, type 2 diabetic patient myocardial mitochondria showed an increased sensitivity to Ca^2+^-induced MPT pore opening, accompanied by a greater rate of mitochondrial H_2_O_2_ emission and 25 % reduction in mitochondrial Ca^2+^ (mCa^2+^) retention capacity compared to nondiabetic patients [36]. Similarly, MPT pore opening in isolated heart mitochondria from Zucker Fa/fa rats with type 2 diabetes was more sensitive to Ca^2+^ than that in mitochondria from lean rats [48].

We suggested that type 1 experimental diabetes resulted in a disturbance in calcium homeostasis in the heart mitochondria of rats and the naringin administration improved it. Recently, in our experiment *in vitro* we showed that naringin (75 μM) promoted membrane potential dissipation, partially prevented Ca^2+^-induced cardiac mitochondrial morphological transformations (200 μM) and calcium-induced MPT pore formation, dose-dependently inhibited the respiratory activity (10 to 75 μM) in isolated rat cardiac mitochondria [29].

Multiple factors are involved in the etiology of diabetic heart failure. Cardiac muscle cell apoptosis is one of the essential processes in cardiomyopathy associated with sustained hyperglycaemia and diabetes mellitus, resulting in the loss of cardiac myocytes and contractile units, the reduction of organ function, and the progression of heart failure. Cardiomyocyte apoptosis is an important therapeutic target under cardiomyopathy [49,50].

A lot of data deals with the beneficial effects of naringin in diabetes. However, the underlying mechanisms by which naringin regulates cardiomyocyte functional activity and prevents cellular impairments are not completely understood. It was demonstrated earlier that naringin attenuated high glucose-induced mitochondrial dysfunction in embryonic rat heart cells, increased mitochondrial membrane potential, inhibited the activation of caspase-3, -8 and -9 and the p38 signalling pathway and, this way, inhibited high glucose-induced apoptosis [16]. Similarly, naringin treatment inhibited both the extrinsic and intrinsic pathways of apoptosis in the liver of STZ rats by preventing the expression of Fas/FasL/caspase-3 as well as an increment of Bax/Bcl-2 ratio [51], inhibited pro-apoptotic proteins P53, P16^INK4a^, Wnt/β-catenin, and related apoptosis pathway in nucleus pulposus cells, while the expression of anti-apoptotic proteins was increased [52]. Similarly, naringin significantly improved cell survival, downregulated cytochrome P450 2E1 (CYP2E1), mitigated the stimulation of oxidative stress, and rectified the antioxidant protein contents of the Nrf2 factor, demonstrating the antioxidant, anti-inflammatory and anti-apoptotic effects [53].

On the other hand, one of the mechanisms of the therapeutic effect of naringin may be apoptosis induction. It was concluded that naringin promoted gastric carcinoma cell apoptosis via blocking the PI3K/AKT signalling pathway and activating cell autophagy in a dose- and time-dependent manner [54]. Similarly, ROS accumulation, DNA damage and activation of apoptosis-like cell death were observed in *E. coli* after treatment with naringin [55].

It was shown that naringin (20-60 (mg/kg)/day, 4 weeks) exerted cardioprotection in type 2 diabetic (db/db) mice through multiple mechanisms: reduced diastolic [Ca^2+^] overload, improved cardiomyocyte glucose uptake, restored the activities of the mitochondrial tricarboxylic acid cycle and respiratory chain enzymes, decreasing ROS production [19]. Naringin prevented morphological impairments in kidney mitochondria, decreased the activities of Krebs cycle enzymes and ATP synthase in mitochondria from the renal cortex in STZ-treated rats [20], and ameliorated the rat liver damage during STZ-induced diabetes, mitigated inflammation and blocked the iNOS/NO/nitrosylated protein pathway [51].

## Conclusions

In conclusion, type 1 experimental diabetes in rats resulted in decreased oxygen consumption rates by isolated cardiac mitochondria and in an increasing rate of spontaneous swelling of isolated diabetic cardiac mitochondria (without the addition of exogenous calcium), while the initial membrane potential value remained unchanged. We revealed that the effects of calcium ions on the membrane potential dissipation and MPT pore opening in isolated cardiac mitochondria decreased under diabetes in comparison with control, probably due to reduced mitochondrial calcium uptake. The long-term (4-week) naringin treatment (40 mg/kg) of rats during diabetes improved such signs of diabetes as islet architecture disruption, glycated haemoglobin accumulation, hyperproteinaemia and oxidative stress, and partially recovered the diabetes-induced complications: impairments in cardiac mitochondria oxygen consumption and mitochondrial sensitivity to calcium ions. Thus, naringin administration demonstrated beneficial effects during type 1 diabetes and represents a promising therapeutic (or nutriceutical) approach for diabetes treatment.
